# Assessing Nutritional Traits and Phytochemical Composition of Artisan Jams Produced in Comoros Islands: Using Indigenous Fruits with High Health-Impact as an Example of Biodiversity Integration and Food Security in Rural Development

**DOI:** 10.3390/molecules23102707

**Published:** 2018-10-20

**Authors:** Dario Donno, Maria Gabriella Mellano, Saandia Hassani, Marta De Biaggi, Isidoro Riondato, Giovanni Gamba, Cristina Giacoma, Gabriele Loris Beccaro

**Affiliations:** 1 Dipartimento di Scienze Agrarie, Forestali e Alimentari, Università degli Studi di Torino, 10095 Grugliasco, Italy; gabriella.mellano@unito.it (M.G.M.); marta.debiaggi@unito.it (M.D.B.); isidoro.riondato@unito.it (I.R.); giovanni.gamba@unito.it (G.G.); gabriele.beccaro@unito.it (G.L.B.); 2 École National de Cuisine et d’Application-Codcom, 167 Moroni, Comoros; saandiacodcom@gmail.com; 3 Dipartimento di Scienze della Vita e Biologia dei Sistemi, Università degli Studi di Torino, 10123 Torino, Italy; cristina.giacoma@unito.it

**Keywords:** fruit jams, food security, phenolic acids, quercetin, agro-biodiversity, HPLC fingerprint

## Abstract

In the Comoros Islands, as in other developing countries, malnutrition and food insecurity affect a very large percentage of the population. Developing fruit-based products in order to make profit, reduce poverty and improve indigenous people diet could be very important for local population of countries as Comoros Islands. The aim of the present work was to study the chemical composition of jams and jellies produced from seven fruit species harvested in Grand Comore Island. The following parameters were studied sugars and organic acids, total phenolics, total anthocyanins and high-performance liquid chromatography (HPLC) fingerprint of the main phytochemicals. Antioxidant activity was also measured. A multivariate approach (Principal Component Analysis) was performed in order to better characterize the products and to set a potential analytical tool for jam characterisation. Results showed that the analysed products are a good source of polyphenolic constituents, as caffeic and gallic acids, catechin and quercetin and volatile compounds, as limonene and γ-terpinene: these molecules may be considered as suitable markers for these fruit-derived products as characterizing the chromatographic patterns. The characterisation of these products and their nutritional and nutraceutical traits is important as valorisation of local food production for poverty reduction and rural development. Further benefits of this approach include the maintenance of local agro-biodiversity as raw material for fruit-based products and the strengthening of food security practices.

## 1. Introduction

The Comoros Islands are situated off the coast of East Africa (290 km), at the northern entrance of the channel of Mozambique between Madagascar and the southern-east African mainland. The archipelago is composed of four main islands: Grand Comore (Ngazidja), Anjouan (Ndzuani), Mohéli (Mwali) and Mayotte (Maore). Comoros Islands are characterized by high plant biodiversity but less than one-sixth of the land remains covered by forest due to a rapid deforestation mainly caused by domestic firewood consumption [1].

Comoros, one of the world’s poorest countries, has an economy based on subsistence agriculture and fishing. In the Comoros Union, as in all the developing countries, malnutrition and food insecurity are the main challenges: in particular energy, malnutrition in children and micronutrient deficiencies (e.g., vitamin deficiency and nutritional anaemias), are important public health issues influencing productivity, maternal/infant health and intellectual development. The improvement of productivity and post-harvest techniques is pivotal to increase Comorian smallholder farmers’ income in order to help fighting poverty and ensure a medium-high nutrition [2].

On the other hand, an abundance of tropical fruit species, often underexploited, grown in semi-natural conditions occurring in Comoros. Tropical fruits attract special attention as they usually have stronger antioxidant properties than common fruits, thanks to bioactive compounds as polyphenols (anthocyanins, flavonoids, phenolic acids and tannins), carotenoids, organic acids and vitamins (B2, B6, C, E, P, PP), as reported in several studies [3,4]. Health-promoting components occurring in these fruits, in particular polyphenols, also show anti-microbial, anti-carcinogenic and anti-viral effects [5]. Therefore, tropical fruits show a high considerable horticultural and nutritional importance for the diet of the rural population [6] in specific critical periods of the year providing sustenance to millions of people. Unfortunately, a significant amount (30–40%) of total fruit production in developing countries is wasted due to poor postharvest handling and inadequate marketing and/or storage facility [7]. Moreover, most of population in these countries lives in rural areas and depends on subsistence agriculture for their livelihoods. In these settlements, agriculture is dependent on rainfall. Inadequacy of food during the dry season and in early summer before the harvest period exposes people to inadequate intake of both macro- and micro-nutrients [6].

Fruit processing industries making preserves, jams, sauces and jellies play an important role in reducing these losses of fruit production, thriving largely as a domestic-product market [8]. In particular, some of the most popular postharvest-stable products made from fruit are jams and jellies, both at the household and commercial levels. Jams and jellies are defined as a mixture, brought to a suitable gelled consistency, of sugars, pulp of one or more fruits and water [9]. To manufacture these products, fruits and sugars are combined in a similar ratio, followed by cooking, to produce a tasty product of sufficiently high sugar content with satisfactory keeping qualities [10]. Jam processing is an important strategy to preserve perishable fruits and improve food security in developing countries [11]. 

Considering new potential agro-industrial and commercial activities related to jam and jelly production, including value-added product labelling, it is of essential importance to guarantee both high quality and compliance with the product specification. For these purposes, increasingly sophisticated analytical methodologies, based on chemical markers, have been developed. Chemical fingerprint methods include the analysis of organic acids, pigments (as carotenoids and anthocyanins), sugars, phenolics and other bioactive compounds [12]. In particular, secondary plant metabolites are very suitable as chemotaxonomic markers [13]. Quantitative differences may occur depending on fruit genotype (e.g., species and cultivar), maturity stages and environmental growth [14], storage conditions [15] and on presence of the skin in fruit-based products [16]. In previous research, they have been successfully used for the determination of the adulteration of some fruit jams and jellies as reported by Dragovic-Uzelac et al. [17].

The aim of the present work was to study chemical compositions of jams and jellies from seven tropical fruit species, harvested near Moroni in Comoros Islands. The following parameters were studied: sugar and organic acid contents, total phenolics (TPC), total anthocyanins (TAC) and fingerprint of the main phytochemicals with demonstrated health-promoting activity by high-performance liquid chromatography (HPLC). Furthermore, antioxidant activity was measured in these products. A multivariate approach (Principal Component Analysis—PCA) was performed in order to better characterize fruit-based products and set a potential analytical tool for analysis and characterisation of local jams and jellies. This study could contribute to the commercial valorisation of these fruit-derived products in rural communities in Comoros Islands thereby reducing post-harvest losses, promoting food security, enhancing small farmers’ income and contributing to a sustainable rural development. 

## 2. Results and Discussion

### 2.1. Nutraceutical Properties

Food processing plays an important role in the bioactive compound degradation, because several transformations of phenolics occur to produce yellowish or brownish pigments [18]: the final product outward appearance is a crucial in determination of consumers’ choices and anthocyanins are the main food colorants responsible for intense colour (associated with raw material freshness and good quality) [19]. Moreover, physical and biological factors as temperature increase and enzymatic activity may be very important in degradation of polyphenolic compounds [10]. 

In this research, the used methods allowed a rapid measurement of TAC and TPC: the Folin-Ciocalteu method suffers many interferences but it can be a complementary technique applied to confirm and support chromatography results, as reported in this study. The TPC ranged from 10.98 ± 2.18 mg_GAE_/100g_Pr_ (mango jam, CM2) to 625.34 ± 67.86 mg_GAE_/100g_Pr_ (red guava jam, CM8), while TAC ranged from 0.59 ± 0.25 mg_C3G_/100g_Pr_ (mango jam, CM2) to 9.56 ± 0.46 mg_C3G_/100g_Pr_ (orange jam, CM7): values obtained from the analysed extracts (Table 1) were higher than values reported by Poiana et al. [20] and Rababah et al. [21]; the differences in phenolic and anthocyanin content could be due to the effects of several internal and external factors on plant material (genetic variability, climatic conditions and environmental factors) [22]. In particular, Comoros pedoclimatic conditions such as volcanic soil, high temperatures, well distributed rains, influence polyphenolic content in fresh fruits and related products.

Bioactive compound redox properties allow them to act as reducing agents, hydrogen donators and singlet oxygen quenchers [23]. In this research, the Ferric Reducing Antioxidant Power (FRAP) assay was used to evaluate antioxidant capacity of fruit jams and jellies, studying the ability of antioxidants to reduce Fe^3+^ ions to Fe^2+^ ions. Jams and jelly FRAP value ranged from 4.71 ± 2.07 mmol Fe^2+^ kg_Pr_^−1^ (mango jam, CM1) to 25.50 ± 0.28 mmol Fe^2+^ kg_Pr_^−1^ (red guava jam, CM8) as shown in Table 1, in accordance with other studies [24,25]. 

Antioxidant activity of fruit-derived products was determined by different bioactive molecules (e.g., polyphenols, as anthocyanins and vitamin C) [26]; for example, antioxidant activity of cyanidin is about 4 times higher compared to ascorbic acid [27]. In this study, the TPC/TAC/antioxidant activity correlation was positive: results showed a significant Pearson correlation coefficients (R = 0.6636 for TPC/antioxidant activity and R = 0.4347 for TAC/antioxidant activity).

### 2.2. Phytochemical Composition

Antioxidant (polyphenols and vitamin C) and anti-inflammatory (terpenes) compounds are the main biologically active substances in fresh fruits and derived products: synergistic or additive health-promoting effects of different phytochemicals (phytocomplex) contribute to biological activity better than a single molecule or a group of few compounds [28]. In this study 22 biologically active compounds together with 9 nutritional substances were selected as markers for HPLC fingerprinting due to their importance in humans [29]. In Supplementary material (Suppl. Figure S1) HPLC chromatograms of orange jams are reported as an example of analysed fruit-based products.

An important question in HPLC analysis is whether the peak comprises one or more components. In quality control and research analysis, impurities hidden behind the peak of interest can falsify results and an undetected component might lead to a loss of essential information. In this research, a peak purity check was assessed in order to control if peaks were pure or contained impurities comparing spectra recorded during the elution of each peak. No-coeluting peaks were detected. Moreover, HPLC-DAD does not allow a definitive identification of phytochemicals. Indeed, liquid chromatography (LC) coupled to mass (MS) or mass/mass spectrometry (MS^2^) is one of the most effective technique for analysis on complex plant extract/fresh fruit/derived product providing a rapid and accurate identification of phytochemicals, as phenolics. For this reason, future developments are necessary but in this preliminary study HPLC-DAD was a simply, rapid and effective approach to describe considered samples in relation to the research aim. Additional markers with demonstrated biological activity could be also taken into consideration for a better identification of the chromatographic pattern of fruit-derived products, together with a mass spectrometry detection of unknown peaks.

The chemical fingerprint of analysed jams and jellies is reported in Table 2, Table 3 and Table 4 (phenolics, other health-promoting components and nutritional substances, respectively). The total bioactive compound content (TBCC) was calculated as the sum of the main molecules (polyphenols, monoterpenes and vitamin C) selected for their biological proved effects on humans and detected in the extracts: TBCC value ranged from 94.25 ± 4.13 mg/100 g_Pr_ (banana flower, CM4) to 357.50 ± 2.10 mg/100 g_Pr_ (orange jam, CM7). This is only a preliminary study on tropical fruit-based jams: in further fingerprint studies, other markers with health-promoting capacity or positive nutritional value should be added for a complete chromatographic pattern characterisation coupling a mass spectrometry (MS) detection of unknown peaks to a UV-visible determination.

**Figure 1:**
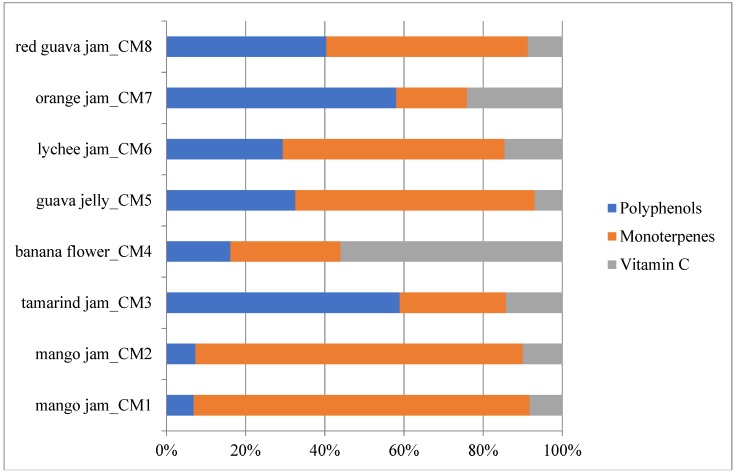
Phytocomplex representation of analysed fruit-derived products. The mean value of each analysed sample is given (*N* = 3).

In Figure 1 identified health-promoting substances were grouped into bioactive classes for the evaluation of the single contribution of each class to total phytocomplex/TBCC (mean values were considered). The most important class in tamarind (CM3) and orange (CM6) jams was polyphenols (58.93% and 58.07%, respectively), expressed as the sum of anthocyanins, phenolic acids, flavanols, catechins and tannins, while monoterpenes were the first class in mango jam (83.79%), guava jelly (60.48%) and lychee jam (56.04%). Banana flower showed a high percentage of vitamin C (56.05%), while red guava jam presented similar content of polyphenols and monoterpenes (40.37% and 50.93%, respectively) with a positive percentage of vitamin C (8.70%).

**Figure 2:**
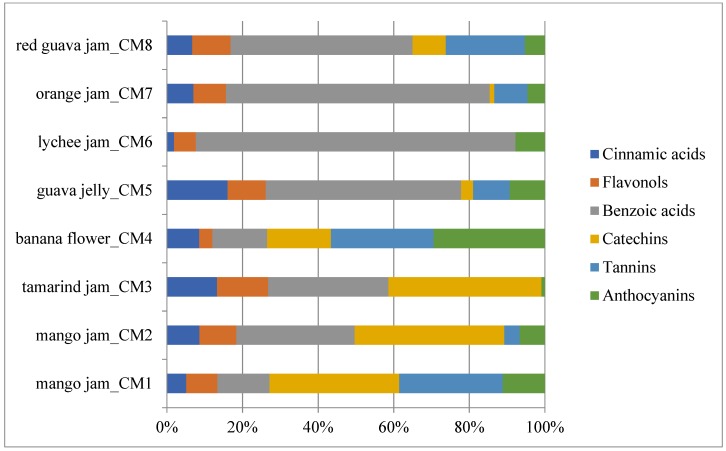
Polyphenolic phytocomplex representation of analysed fruit-derived products. The mean value of each analysed sample is given (*N* = 3).

Results showed that analysed fruit jams and jellies are a very good source of polyphenolic compounds. Figure 2 reports the single contribution of each polyphenolic class to total polyphenols detected by HPLC. Catechins were the most important class in mango and tamarind jams (36.93% and 40.53%, respectively), while phenolic acids (cinnamic acids plus benzoic acids) were the most important classes in guava jelly (67.75%) and in lychee (86.46%), orange (76.77%) and red guava (54.82%) jams. Banana flower showed a high percentage of tannins and anthocyanins (27.21% and 29.42%, respectively), followed by catechins (16.81%) and benzoic acids (14.53%). 

Analysed jams and jellies presented interesting quali-quantitative polyphenolic profiles if compared to commercial products derived from common temperate fruits; in particular, they showed higher TPC values and relative antioxidant activity than strawberry (101.40 mg_GAE_/100 g_Pr_) [25], apricot (51.49 mg_GAE_/100 g_Pr_) [21], berry fruits (336.67 mg_GAE_/100 g_Pr_) [24], peach (18.85 mg_GAE_/100 g_Pr_) and apple (20.07 mg_GAE_/100 g_Pr_) [16]. Moreover, jams from tropical fruits showed higher values of specific phenolic markers as coumaric, caffeic and ferulic acids (lychee, banana and guava), catechin (tamarind) and rutin (mango) than commercial products derived from berry fruits (the content values in berry fruits were 0.39 mg/100 g_Pr_ for coumaric acid, 1.38 mg/100 g_Pr_ for caffeic acid, 0.13 mg/100 g_Pr_ for ferulic acid, 3.93 mg/100 g_Pr_ for catechin and 0.26 mg/100 g_Pr_ for rutin) [19]. These results may contribute to better valorise the products derived from local biodiversity compared to imported commercial ones and improve food industry in the Comoros Islands with the potential exportation of these productions.

In some studies, phenolic compound characterisation was mainly used for chemotaxonomic purposes; moreover, some researchers reported that fruit processing during jam/jelly production did not change much the qualitative polyphenolic profile [10,30]. In this research, each product showed a specific phenolic composition characterised by the presence of one or more specific markers. Chlorogenic acid proved to be characteristic of tamarind jam (11.45 ± 0.73 mg/100 g_Pr_), guava jelly (13.77 ± 0.55 mg/100 g_Pr_) and orange jam (12.97 ± 0.19 mg/100 g_Pr_) and it could be used as a marker to prove the addition of these fruits to other jams. Moreover, chlorogenic acid is considered a preferential substrate for the catecholase activity of polyphenol oxidase and it may be important during fruit processing [31]. Caffeic acid was detected in all the fruit-derived products in a close range between 0.49 ± 0.13 mg/100 g_Pr_ (tamarind jam, CM3) and 0.76 ± 0.12 mg/100 g_Pr_ (lychee jam, CM6) similar to other studies [32,33]. Coumaric acid was the most important cinnamic acid in red guava jam-CM8 (5.58 ± 0.80 mg/100 g_Pr_), while ferulic acid was present in tamarind jam-CM3 (1.38 ± 0.22 mg/100 g_Pr_) and orange jam-CM7 (1.00 ± 0.11 mg/100 g_Pr_) as reported by Jimohand Onabanjo [34] and Marquina et al. [35]. Caffeic, ferulic and coumaric acids could be involved in the oxidation processes and colour development during technological processing [36]. Flavonoids (flavanols and catechins) were also demonstrated to be important as markers for orange and tamarind jam quality control [37], because flavonoids are not affected by the manufacturing process. Flavonols quench active oxygen species [38] and inhibit in vitro oxidation of low-density lipoproteins reducing thrombotic tendency. In this research, the most effective flavonol selected as marker was quercetin in guava jelly (7.56 ± 0.03 mg/100 g_Pr_) and jams of tamarind (11.44 ± 0.96 mg/100 g_Pr_), orange (15.69 ± 1.11 mg/100 g_Pr_) and red guava (7.62 ± 0.20 mg/100 g_Pr_), while rutin proved to be characteristic of mango jams (0.94 ± 0.04 mg/100 g_Pr_ for CM1 and 0.86 ± 0.03 mg/100 g_Pr_ for CM2) according to other studies [39,40]. The identification of catechin (maximum value of 40.64 ± 1.56 mg/100 g_Pr_ in tamarind jam) and epicatechin (maximum value of 4.03 ± 0.16 mg/100 g_Pr_ in red guava jam) could be useful: indeed, they are involved in the lipid peroxidation inhibition and human cancer cell line proliferation as other similar compounds [41]. The presence of tannins in adequate amounts in orange and red guava jams (18.24 ± 2.52 mg/100 g_Pr_ and 19.79 ± 0.50 mg/100 g_Pr_, respectively) are positive as they are free radical quenchers [42]. High levels of ellagic acid in tamarind jam (23.30 ± 1.96 mg/100 g_Pr_) and orange jam (57.11 ± 1.64 mg/100 g_Pr_) as well as high content of gallic acid in guava jelly (42.02 ± 0.58 mg/100 g_Pr_) and orange jam (87.72 ± 1.81 mg/100 g_Pr_) were also detected: these molecules are endowed with numerous biological properties, as anticancer, anti-inflammatory and anti-HIV replication activities [43]. These preliminary results on phenolic composition demonstrate the need of identifying more bioactive substances for control of the authenticity of fruit-based products. 

Anthocyanins have frequently been also considered for the cited purposes, because their specific patterns may allow the classification of fruit species and relative derived products and the characterization of their nutraceutical and nutritional traits (e.g., detection of admixtures of fruits with a more stable colour during jam processing). As previously discussed, anthocyanins are of prominent importance in guava jelly (8.35 ± 1.13 mg_C3G_/100g_Pr_), orange jam (9.56 ± 0.46 mg_C3G_/100g_Pr_) and red guava jam (4.99 ± 0.67 mg_C3G_/100g_Pr_) because i) they are important for quality traits, due to their levels directly related to the product colour and ii) they have been proved to show several health-promoting activities and a high potential phytochemical value [44]. As opposed to other polyphenolic compounds, composition in anthocyanins may be subject to modification during processing and storage steps as reported by Garzon and Wrolstad [45]: in particular, enzymation during jam processing may change anthocyanin patterns [46]. During jam and jelly storage new pyranoanthocyanins may also be formed by direct reaction of anthocyanins with cinnamic acids, as shown by Schwarz et al. [47]. For this reason, anthocyanins may be only used as quantitative markers in the quality control of jams and similar products.

Monoterpenes represent an important fraction of the TBCC in analysed fruit-based products, in particular limonene in mango jam (103.53 ± 3.12 mg/100 g_Pr_) and guava jelly (102.75 ± 0.58 mg/100 g_Pr_) and γ-terpinene in jams of mango (48.07 ± 2.30 mg/100 g_Pr_) and red guava (43.01 ± 0.16 mg/100 g_Pr_): the plant terpenoids are a large class of phytochemicals used for their aromatic qualities and antioxidant and anti-inflammatory activity [48]. Monoterpenes are non-nutritive dietary substances with antibacterial and antitumor activity found in the essential oils of several plants [49] and several studies reported their chemopreventive activity against several cancers [50]. 

Vitamin C value was obtained as the sum of ascorbic and dehydroascorbic acids due to their biological activity in humans [51]. In this research banana flower and orange jams showed a high vitamin C content (52.82 ± 0.57 mg/100 g_Pr_ and 86.07 ± 0.48 mg/100 g_Pr_, respectively). Other analysed products showed good values of vitamin C (about 10–25 mg/100 g_Pr_) according to previous similar studies [52,53].

**Figure 3:**
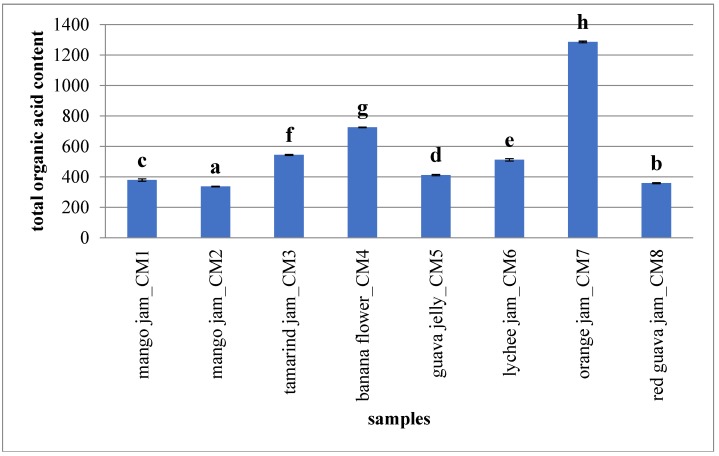
Total organic acid content of analysed fruit-derived products. The mean value of each analysed sample is given (*N* = 3). Different letters for each class indicate the significant differences at *p* < 0.05. Results are expressed as mg/100 g of product.

Organic acids in fruits are little influenced by changes during processing and storage and show a good stability if compared to pigments and flavour compounds. Accordingly, their identification may be suitable for the estimation of fruit amount and for the fruit quality control [54]. However, since organic acids (e.g., citric acid) are indispensable technological components of most derived-products, they are not applicable as quality control markers in fruit jams and jellies. Furthermore, organic acid composition is influenced by genetic factors (e.g., cultivar) and degree of ripeness, limiting their applicability as a quantitative marker in fruit-derived products [55]. In any case, they are important antioxidants with multi-purpose uses in pharmacology as reported by Eyduran et al. [56]. They were also utilised as food acidifiers by food companies [57]. In this research orange jam showed high variability in organic acid composition: in particular, succinic acid (406.65 ± 4.40 mg/100 g_Pr_) and quinic acid (391.13 ± 2.19 mg/100 g_Pr_) were found to be suitable markers for jam characterization because they were not detected in other analysed fruit-based products and represent specific molecules of orange jam chromatographic pattern as shown by Cejudo-Bastante et al. [58] and Flores et al. [59]. Similarly, malic acid could be a specific marker for mango jams and tartaric acid for guava jelly and jams of lychee and red guava. Figure 3 shows total organic acid content in analysed fruit-based products.

**Figure 4:**
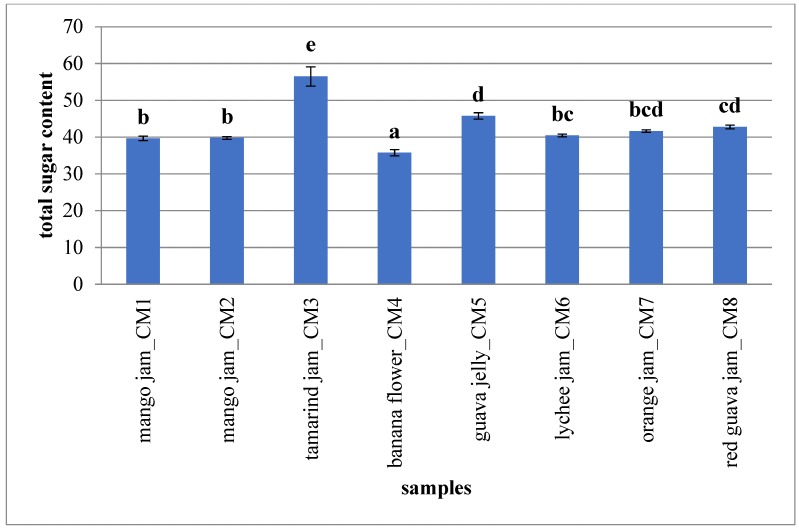
Total sugar content of analysed fruit-derived products. The mean value of each analysed sample is given (*N* = 3). Different letters for each class indicate the significant differences at *p* < 0.05. Results are expressed as g/100 g of product.

Apart from organic acids, the sugar pattern was also utilised for fruit species differentiation, while their use as quantifiers of fruit content is very limited. Identical sugar profiles, used often for the detection of illegal adulterations as the admixture of sugar solutions or fruit juices, were observed for fruits from different countries as well as for different genotype [55]. In this research sugar pattern (Figure 4) was studied in order to evaluate the nutritional potential of analysed fruit-derived products. Tamarind jam showed the highest sugar content (56.50 ± 2.70 g/100 g_Pr_), expressed as sum of fructose (15.35 ± 0.14 g/100 g_Pr_), glucose (27.64 ± 2.33 g/100 g_Pr_) and sucrose (13.51 ± 0.23 g/100 g_Pr_). Other analysed products presented a total sugar content of about 40 g/100 g_Pr_ as reported in other studies [60,61].

### 2.3. Multivariate Analysis

**Figure 5:**
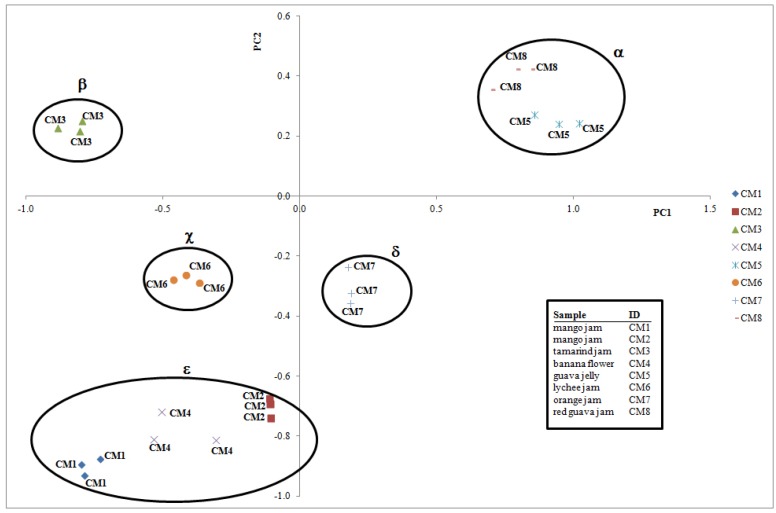
PCA score plot of fruit-derived products (eight samples and three replications per sample). The ellipses around each object group only indicate the position of a category in the plot without statistical meaning, based on the phytochemical results.

Single markers of the same phytochemical group were combined in bioactive classes for multivariate data handling. For the visualisation of potential differences in the products and easily characterize analysed jams and jellies, PCA was performed on all the data and it reduced the initial variables (TPC, antioxidant activity, TAC, content of 9 chemical classes) to three principal components (86.74% of total variance), placing the eight fruit-based products in the PCA score plot (Figure 5) in relation to phytochemical composition, nutritional properties and nutraceutical traits. PC1 and PC2 well represent the system information (70.20% of total variance); the PCA results showed five groups, highlighted in Figure 5 with circles, without statistical meaning, according to the phytochemical results; the groups were named α (guava jelly—CM5, red guava jam—CM8), β (tamarind jam—CM3), χ (lychee jam—CM6), δ (orange jam—CM7) and ε (mango jam, genotype 1—CM1, mango jam, genotype 2—CM2, banana flower—CM4). 

**Figure 6:**
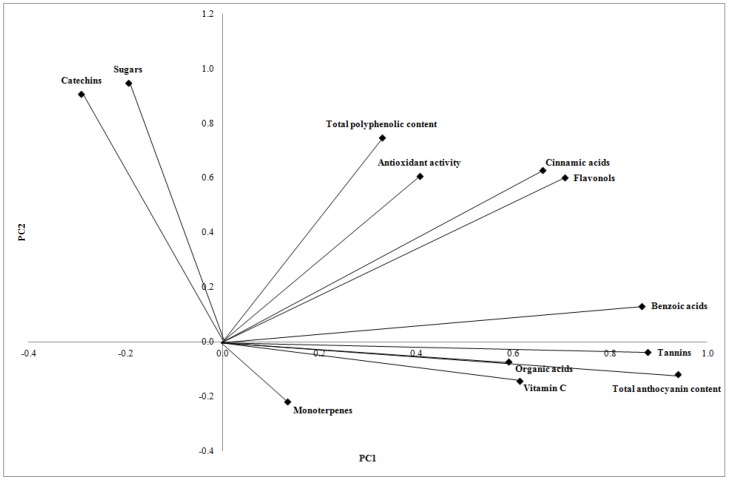
PCA loading plot of fruit-based products showing correlation among nutritional traits, nutraceutical properties and bioactive compound classes and PCs.

PCA loading plot showed a correlation between most of the polyphenolic classes (phenolic acids, tannins, anthocyanins and flavanols), vitamin C, organic acids and PC1 (43.15% of total variance) and a correlation between TPC, catechins, antioxidant activity, sugars and PC2 (27.05% of total variance). Monoterpenes showed an intermediate position between PC1 and PC2 (Figure 6). Polyphenolic compounds (in particular, benzoic acids, tannins and anthocyanins) and vitamin C presented a high discriminating power among different samples, as reported in other studies [37,55], as well as catechins.

These results showed that PCA classification obtained from main bioactive groups, nutritional traits and nutraceutical properties characterized the analysed products according to the different chemical pattern and provided information on the phytochemical markers with the most influence on the phytocomplex. A chemometric method was applied coupled to an HPLC fingerprint technique for a better recognition of analysed products as reported by Tzouros and Arvanitoyannis [62]. Different marker compounds were found to be the most discriminating variables, which could be applied to accurate composition control of fruit jams and jellies; in particular, the phytocomplex graphical view showed that genotypes included in the α and δ PCA group (guava jelly/red guava jam and orange jam, respectively) present the highest amount of antioxidant compound classes (polyphenols and vitamin C) together with the highest amount of volatile molecules (organic acids and monoterpenes), the most responsible compounds for the product aroma. The combination of chromatographic fingerprinting and chemometrics could be an effective potential tool for quality control of fruit-based products avoiding potential voluntary or involuntary adulterations and contaminations [63]. The analysis of other samples from different origin and nature are required to ensure that the proposed methodology is applicable to the authentication of jellies and jams from Comoros Islands or to the detection of their adulteration. In the present research, this methodology only showed that jams from Comoros Islands may be differentiated among them in relation to their fruit phytochemical composition. Therefore, further additional experiments are required for demonstrating that this methodology can be used for adulteration detection and/or authentication. These hyphenated techniques could also be positively used for the evaluation and differentiation of several products in local markets setting a potential tool to obtain label certifications for the valorisation of local productions.

## 3. Materials and Methods

A detailed description of the used extraction protocols and analytical methods is reported in the Supplementary material.

### 3.1. Plant. Material

The investigated material consisted of fruit-derived products prepared from 7 species (*Mangifera indica* L.—two genotypes, *Tamarindus indica* L., *Musa* × *paradisiaca* L., *Psidium cattleyanum* Sabine, *Litchi chinensis* Sonn., *Citrus sinensis* (L.) Osbeck and *Psidium guajava* L.). Fruits were picked at commercial maturity stage during the 2017 from commercial orchards managed by the *Coopérative et Mutuelle des Comores pour le Développement* (Codcom) in Moroni, Comoros Islands. CODCOM is a non-governmental organization involved in poverty reduction in Comoros and in the countries of the Indian Ocean. Physiologically mature fruits were randomly selected from three plants for each biological replication (*n* = 3). Fruits were manually harvested from the plants based on selected qualitative parameters (e.g., colour, firmness and total soluble solids), considering also literature and experience of the local researchers and technicians. Fruits were then sorted, washed and stored in a 4 °C cold room for less than 5 days until jam and jelly preparation. Inedible parts were rejected.

### 3.2. Preparation of Fruit-Derived Products

A standard commercial procedure to manufacture jams and jellies was followed.

Fruit (1 kg) was reduced to fine particles with a commercial food processor for approximately 30 s. Jam formulation was 50% fruit, 48% sugar and 2% pectin mix (composed of dextrose, pectin and fumaric acid). pH was checked and adjusted if needed by addition of 50% citric acid solution (10–15 mL) to a target pH of 3.0. An essence of vanilla or cinnamon was added to improve the sensory properties of the final products. Sugar was added and the mixture was boiled for 30–40 min to a final concentration of 65°Brix (approximately 105 °C final boiling point). The jam was packed at 90 °C in 350 mL glass jars, immediately sealed with plastisol lined metal lids and inverted for 5 min to sterilize the lids. The jars were then returned to normal position for air-cooling.

For the preparation of 1 kg fruit jelly, 500 g filtered fruit juice, 550 g sugar, 5 g citric acid was used. Five g of pectin were added. The remaining sugar was mixed with fruit juice and heated until the total soluble solid (TSS) become near to 55°Brix. Then sugar mixed pectin was added and continued the heating until TSS becomes near to 58°Brix. The citric acid was added and continued the heating. When TSS of the jelly becomes 67°Brix, jelly was poured in a sterilized glass bottle and parafinning the cap.

### 3.3. Spectrophotometric Analysis

Total polyphenolic content (TPC) was determined according to the Folin-Ciocalteu colorimetric method [64,65]; results were expressed as mg of gallic acid equivalents (GAE) per 100 g of product (Pr). 

Total anthocyanin content (TAC) was determined using the pH-differential method [66,67] and expressed as milligrams of cyanidin-3-*O*-glucoside (C3G) per 100 g of product (mg_C3G_/100 g_Pr_).

Antioxidant activity was evaluated by Ferric Reducing Antioxidant Power (FRAP) assay [68], with the results expressed as millimoles of ferrous iron (Fe^2+^) equivalents per kilogram (solid food) of Pr.

### 3.4. Chromatographic Analysis

Chromatographic analysis was carried out using an Agilent 1200 high-performance liquid chromatograph coupled to an Agilent UV-Vis diode array detector (Agilent Technologies, Santa Clara, CA, USA), based on HPLC methods previously validated for fresh fruits, herbal medicines and other food products [2,69].Composition of solvents, used gradient elution conditions and UV-Vis wavelengths were listed and described in Table 5, while calibration parameters for all the used analytical standards were reported in Table 6 [70]. 

### 3.5. Statistical Analysis

Uni- and multivariate analysis (MVA) were carried out on all of the samples. Data were treated by one-factor analysis of variance (ANOVA) and the averages were compared with Tukey’s HSD post-hoc comparison test at significance level *p* < 0.05 (*N* = 3). Correlation between antioxidant activity and TPC/TAC was evaluated with Pearson’s coefficient (R) at *p* < 0.05 (*N* = 3). For discrimination of the investigated samples, principal component analysis (PCA) was performed on the column-centred data. All calculations were performed with statistical software package IBM SPSS Statistics 22.0 (IBM, Armonk, NY, USA).

## 4. Conclusions

In this study, spectrophotometric and chromatographic methods coupled to chemometrics were used for phytochemical analysis in order to detect and quantify bioactive substances and characterize nutraceutical properties and nutritional traits in fruit-based products from Comoros Islands. HPLC profile of phenolic compounds may be used as ‘‘fingerprint’’ in the detection ofquali-quantitative differences in jellies and jams.

Results showed that analysed fruit jams and jellies could be a good source of polyphenolic constituents, as benzoic and cinnamic acids, catechins and flavanols and volatile compounds, as organic acids and monoterpenes: these molecules were found to be suitable markers for product characterization because they were specific compounds of the obtained chromatographic patterns. For this reason, even if each analytical approach has its limitations which restrict its applicability, the identification of these chemical markers could be a simple, rapid and generally available potential tool for quality control and labelling of local jams and jellies.

Moreover, the characterisation of these products and their nutritional and nutraceutical traits could be important to valorise local food production and to raise incomes for local population in the Comoros Islands, particularly women that work in agri-food industry. The obtained incomes could be useful to reduce poverty; indeed, the advances in local food production can be important in poverty reduction and deserves greater attention in rural development: it is necessary to link the evidence of poverty impact to simple policy recommendations in order to potentially integrate the promotion of local fruit-based products into national-level planning. 

Finally, further benefits of this approach include the potential for a better nutrition, maintenance of biodiversity and environmentally sustainable food systems.

## Figures and Tables

**Table 1:** Nutraceutical traits of the analysed fruit-derived products.

		Total Polyphenolic Content			Antioxidant Activity			Total Anthocyanin Content		
		(mg_GAE_/100 g_Pr_)			(mmol Fe^2+^/kg_pr_)			(mg_C3G_/100 g_Pr_)		
Sample	ID	Mean Value	SD	Tukey Test	Mean Value	SD	Tukey Test	Mean Value	SD	Tukey Test
mango jam	CM1	11.11	1.34	a	4.71	2.07	a	1.48	0.26	ab
mango jam	CM2	10.98	2.18	a	7.86	0.36	b	0.59	0.25	a
tamarind jam	CM3	484.95	81.89	d	23.97	0.28	d	0.87	0.43	ab
banana flower	CM4	13.06	1.85	a	19.23	1.29	c	4.49	1.62	c
guava jelly	CM5	437.13	2.44	d	25.40	0.35	d	8.35	1.13	d
lychee jam	CM6	266.96	7.95	c	23.01	0.07	d	3.15	0.66	bc
orange jam	CM7	124.50	27.49	b	16.62	0.89	c	9.56	0.46	d
red guava jam	CM8	625.34	67.86	e	25.50	0.28	d	4.99	0.67	c

Mean value and standard deviation (SD) of each sample is given (*N* = 3). Different letters for each class indicate the significant differences at *p* < 0.05. GAE = gallic acid equivalent; C3G = cyanidin 3-*O*-glucoside. Pr = product.

**Table 2:** Phytochemical fingerprint of the polyphenolic compounds in analysed jams and jellies.

		Mango Jam, CM1		Mango Jam, CM2		Tamarind Jam, CM3		Banana Flower, CM4		Guava Jelly, CM5		Lychee Jam, CM6		Orange Jam, CM7		Red Guava Jam, CM8	
		Mean Value	SD	Mean Value	SD	Mean Value	SD	Mean Value	SD	Mean Value	SD	Mean Value	SD	Mean Value	SD	Mean Value	SD
Cinnamic acids	caffeic acid	0.678	0.170	0.761	0.101	0.498	0.134	0.749	0.154	0.687	0.034	0.764	0.116	0.571	0.110	0.744	0.204
	chlorogenic acid	n.d.	/	n.d.	/	11.454	0.731	n.d.	/	13.766	0.546	n.d.	/	12.971	0.185	n.d.	/
	coumaric acid	n.d.	/	n.d.	/	n.d.	/	0.556	0.509	n.d.	/	n.d.	/	n.d.	/	5.583	0.797
	ferulic acid	n.d.	/	n.d.	/	1.377	0.217	n.d.	/	n.d.	/	n.d.	/	1.001	0.110	n.d.	/
Flavonols	hyperoside	n.d.	/	n.d.	/	n.d.	/	0.356	0.033	n.d.	/	1.027	0.116	n.d.	/	n.d.	/
	isoquercitrin	n.d.	/	n.d.	/	0.491	0.006	n.d.	/	0.525	0.034	n.d.	/	1.140	0.110	0.541	0.155
	quercetin	n.d.	/	n.d.	/	11.441	0.963	n.d.	/	7.562	0.034	n.d.	/	15.694	1.107	7.621	0.204
	quercitrin	0.161	0.030	0.007	0.003	1.640	0.257	0.173	0.019	1.001	0.034	1.334	0.116	1.152	0.110	1.477	0.256
	rutin	0.936	0.036	0.858	0.034	n.d.	/	n.d.	/	n.d.	/	n.d.	/	n.d.	/	n.d.	/
Benzoic acids	ellagic acid	1.167	0.264	1.228	0.154	23.302	1.962	1.604	0.365	4.444	0.316	3.799	0.116	57.105	1.637	4.561	0.607
	gallic acid	0.668	0.105	1.535	0.232	8.571	0.314	0.611	0.070	42.023	0.582	30.650	0.886	87.724	1.812	40.955	1.727
Catechins	catechin	0.872	0.023	1.749	0.175	40.644	1.558	1.216	0.162	0.327	0.034	n.d.	/	0.436	0.110	4.287	0.204
	epicatechin	3.683	0.328	1.747	0.400	n.d.	/	1.348	0.184	2.523	0.034	n.d.	/	2.015	0.110	4.031	0.157
Tannins	castalagin	n.d.	/	n.d.	/	n.d.	/	2.595	0.425	4.384	0.034	n.d.	/	15.210	2.414	9.773	0.295
	vescalagin	3.639	0.174	0.359	0.008	n.d.	/	1.554	0.371	4.339	0.205	n.d.	/	3.031	0.110	10.012	0.204

Mean value and standard deviation (SD) of each sample is given (*N* = 3). Results are expressed as mg/100 g_Pr_. Pr = product. n.d. = not detected.

**Table 3:** Phytochemical fingerprint of monoterpenes and vitamin C in analysed jams and jellies.

		Mango Jam, CM1		Mango Jam, CM2		Tamarind Jam, CM3		Banana Flower, CM4		Guava Jelly, CM5		Lychee Jam, CM6		Orange Jam, CM7		Red Guava Jam, CM8	
		Mean Value	SD	Mean Value	SD	Mean Value	SD	Mean Value	SD	Mean Value	SD	Mean Value	SD	Mean Value	SD	Mean Value	SD
Monoterpenes	limonene	103.529	3.116	31.201	0.588	n.d.	/	n.d.	/	102.750	0.582	33.602	0.687	18.818	0.472	51.319	3.204
	phellandrene	27.843	2.380	7.809	0.540	7.497	0.219	n.d.	/	4.671	0.034	4.668	0.116	n.d.	/	4.946	0.204
	sabinene	8.140	0.045	12.187	1.146	11.273	1.180	n.d.	/	12.847	1.020	12.811	0.687	n.d.	/	12.332	0.797
	γ-terpinene	24.552	2.464	48.067	2.301	n.d.	/	17.949	2.097	39.002	0.034	19.069	0.492	37.084	0.505	43.005	0.155
	terpinolene	n.d.	/	n.d.	/	26.944	2.718	8.228	0.213	7.731	0.034	7.473	0.886	7.926	0.110	7.715	0.564
Vitamin C	ascorbic acid	15.058	0.362	10.178	0.072	20.397	0.117	49.699	0.356	18.074	0.138	18.978	0.057	54.062	0.074	19.104	0.487
	dehydroascorbic acid	0.813	0.130	1.815	0.163	3.767	0.283	3.123	0.213	1.138	0.192	1.196	0.225	32.003	0.401	1.279	0.234

Mean value and standard deviation (SD) of each sample is given (*N* = 3). Results are expressed as mg/100g_Pr_. Pr = product. n.d. = not detected.

**Table 4:** Phytochemical fingerprint of nutritional substances in analysed jams and jellies.

		Mango Jam, CM1		Mango Jam, CM2		Tamarind Jam, CM3		Banana Flower, CM4		Guava Jelly, CM5		Lychee Jam, CM6		Orange Jam, CM7		Red Guava Jam, CM8	
		Mean Value	SD	Mean Value	SD	Mean Value	SD	Mean Value	SD	Mean Value	SD	Mean Value	SD	Mean Value	SD	Mean Value	SD
Organic acids	citric acid	16.217	4.058	29.696	0.469	522.583	2.566	694.580	3.627	326.950	3.030	268.422	2.629	287.911	4.614	200.408	2.204
	malic acid	357.878	3.679	302.528	2.531	n.d.	/	21.926	1.696	n.d.	/	n.d.	/	n.d.	/	n.d.	/
	oxalic acid	4.741	0.198	4.573	0.416	21.444	0.530	8.441	0.102	8.194	0.117	n.d.	/	6.629	0.110	23.717	0.797
	quinic acid	n.d.	/	n.d.	/	n.d.	/	n.d.	/	n.d.	/	138.233	1.263	391.128	2.192	n.d.	/
	succinic acid	n.d.	/	n.d.	/	n.d.	/	n.d.	/	n.d.	/	n.d.	/	406.648	4.401	n.d.	/
	tartaric acid	n.d.	/	n.d.	/	n.d.	/	n.d.	/	76.724	1.513	105.358	4.157	193.497	3.661	134.637	1.729
Sugars	fructose	11.701	0.038	11.316	0.100	15.353	0.140	12.275	0.287	21.341	0.034	14.814	0.116	13.620	0.110	14.428	0.204
	glucose	14.505	0.321	13.620	0.401	27.637	2.326	12.342	0.111	12.615	0.034	11.044	0.116	13.090	0.110	13.413	0.155
	sucrose	13.444	0.339	14.831	0.680	13.509	0.231	11.131	1.084	11.801	0.867	14.573	0.492	14.935	0.110	14.904	0.157

Mean value and standard deviation (SD) of each sample is given (*N* = 3). Results are expressed as mg/100g_Pr_. Pr = product. n.d. = not detected.

**Table 5:** Chromatographic conditions of the used methods.

Method	Compounds of Interest	Stationary Phase	Mobile Phase	Flow (mL min^−1^)	Wavelength (nm)
A	cinnamic acids, flavanols	KINETEX-C18 column (4.6 × 150 mm, 5 μm)	A: 10 mM KH_2_PO_4_/H_3_PO_4_, pH = 2.8	1.5	330
			B: CH_3_CN		
B	benzoic acids, catechins,	KINETEX-C18 column (4.6 × 150 mm, 5 μm)	A: H_2_O/CH_3_OH/HCOOH (5:95:0.1 *v*/*v*/*v*), pH = 2.5	0.6	280
	tannins		B: CH_3_OH/HCOOH (100:0.1 *v*/*v*)		
C	monoterpenes	KINETEX-C18 column (4.6 × 150 mm, 5 μm)	A: H_2_O	1.0	210, 220,
			B: CH_3_CN		235, 250
D	organic acids	KINETEX-C18 column (4.6 × 150 mm, 5 μm)	A: 10 mM KH_2_PO_4_/H_3_PO_4_, pH = 2.8	0.6	214
			B: CH_3_CN		
E	vitamins	KINETEX-C18 column (4.6 × 150 mm, 5 μm)	A: 5 mM C_16_H_33_N(CH_3_)_3_Br/50 mM KH_2_PO_4_, pH = 2.5	0.9	261, 348
			B: CH_3_OH		
F	sugars	SphereClone-NH_2_ column (4.6 × 250 mm, 5 μm)	A: H_2_O	0.5	200, 267,
			B: CH_3_CN		286

*Elution conditions.* Method A: gradient analysis: 5%B to 21%B in 17 min + 21%B in 3 min (2 min conditioning time); Method B: gradient analysis: 3%B to 85%B in 22 min + 85%B in 1 min (2 min conditioning time); Method C: gradient analysis: 30%B to 56%B in 15 min + 56%B in 2 min (3 min conditioning); Method D: gradient analysis: 5%B to 14%B in 10 min + 14%B in 3 min (2 min conditioning time); Method E: isocratic analysis: ratio of phase A and B: 95:5 in 10 min (5 min conditioning time); Method F: isocratic analysis: ratio of phase A and B: 5:85 in 12 min (3 min conditioning time). Chromatographic separation was performed on a Kinetex-C18 column (Phenomenex, Torrance, CA, USA) and a SphereClone-NH2 column (Phenomenex, Torrance, CA, USA).

**Table 6:** Calibration parameters for all the used analytical standards.

Class	Standard	Calibration Curve Equation	R^2^	Calibration Curve Range (mg L^−1^)
Cinnamic acids	caffeic acid	y = 59.046x + 200.6	0.996	111–500
	chlorogenic acid	y = 13.583x + 760.05	0.984	111–500
	coumaric acid	y = 8.9342x + 217.4	0.997	111–500
	ferulic acid	y = 3.3963x − 4.9524	1.000	111–500
Flavonols	hyperoside	y = 7.1322x − 4.583	0.999	111–500
	isoquercitrin	y = 8.3078x + 26.621	0.999	111–500
	quercetin	y = 3.4095x − 98.307	0.998	111–500
	quercitrin	y = 2.7413x + 5.6367	0.998	111–500
	rutin	y = 6.5808x + 30.831	0.999	111–500
Benzoic acids	ellagic acid	y = 29.954x + 184.52	0.998	62.5–250
	gallic acid	y = 44.996x + 261.86	0.999	62.5–250
Catechins	catechin	y = 8.9197x + 66.952	1.000	62.5–250
	epicatechin	y = 12.88x − 43.816	0.999	62.5–250
Tannins	castalagin	y = 4.236x − 8.535	1.000	62.5–250
	vescalagin	y = 4.939x − 1.232	1.000	62.5–250
Monoterpenes	limonene	y = 0.1894x − 5.420	0.999	125–1000
	phellandrene	y = 8.783x − 145.3	0.998	125–1000
	sabinene	y = 18.14x − 1004	0.998	125–1000
	γ-terpinene	y = 0.4886x − 23.02	0.999	125–1000
	terpinolene	y = 26.52x + 876.8	0.999	125–1000
Organic acids	citric acid	y = 1.0603x − 22.092	1.000	167–1000
	malic acid	y = 1.415x − 80.254	0.996	167–1000
	oxalic acid	y = 6.4502x + 6.1503	0.998	167–1000
	quinic acid	y = 0.8087x − 38.021	0.998	167–1000
	succinic acid	y = 0.9236x − 8.0823	0.995	167–1000
	tartaric acid	y = 1.8427x + 15.796	1.000	167–1000
Vitamins	ascorbic acid	y = 42.71x + 27.969	0.999	100–1000
	dehydroascorbic acid	y = 4.1628x + 140.01	0.999	30–300
Sugar	fructose	y = 1.8548x + 1.2324	0.999	125–1000
	glucose	y = 0.1269x − 0.1107	0.998	125–1000
	sucrose	y = 0.296x − 3.2202	1.000	125–1000

Single bioactive molecules were identified and quantified using selected biomarkers with a positive role in human health (“multi-marker approach”) according to Mok and Chau [71]. All the results were expressed as mg/100 g of product (Pr), except sugars (expressed as g/100 g of Pr). Total bioactive compound content (TBCC) were determined as sum of selected compounds and expressed as mg/100 g of product.

## Supplementary Materials

The supplementary materials are available online.
