# Supplementary material for: Assessing Nutritional Traits and Phytochemical Composition of Artisan Jams Produced in Comoros Islands: Using Indigenous Fruits with High Health-Impact as an Example of Biodiversity Integration and Food Security in Rural Development
Source: Molecules. 2018 Oct 20;23(10):2707. doi: 10.3390/molecules23102707 (PMC6222852; doi:10.3390/molecules23102707)
Supplement: Supplementary file 1 [file molecules-23-02707-s001.pdf]

SUPPLEMENTARY MATERIAL

Detailed version of the used extraction protocols and analysis methods

Solvents and chemicals

Sodium carbonate, Folin-Ciocalteu phenol reagent, sodium acetate, citric acid, potassium chloride, hydrochloric acid, iron(III) chloride hexahydrate, 2,4,6-tripyridyl-S-triazine (TPTZ), 1,2-phenylenediamine dihydrochloride (OPDA), all polyphenolic (caffeic acid, chlorogenic acid, coumaric acid, ferulic acid, hyperoside, isoquercitrin, quercetin, quercitrin, rutin, ellagic acid, gallic acid, (+)catechin, (-)epicatechin, castalagin, and vescalagin) and terpenic standards (limonene, phellandrene, sabinene, γ-terpinene, and terpinolene), sugars (fructose, glucose, and sucrose), potassium dihydrogen phosphate, phosphoric acid and HPLC-grade methanol and acetonitrile were purchased from Sigma-Aldrich (St. Louis, MO, USA). Ethylenediaminetetraacetic acid disodium salt was purchased from AMRESCO (Solon, OH, USA). Sodium fluoride was purchased from Riedel-de Haen (Seelze, Germany). Milli-Q ultrapure water was produced by Sartorius Stedim Biotech mod. Arium (Sartorius, Göettingen, Germany). Cetyltrimethylammonium bromide (cetrimide), ascorbic acid (AA) and dehydroascorbic acid (DHAA) were purchased from Extrasynthése (Genay, France). Acetic acid, ethanol, organic acids (citric acid, malic acid, oxalic acid, quinic acid, succinic acid, and tartaric acid) and HPLC-grade formic acid were purchased from Fluka BioChemika, Buchs, Switzerland.

Bioactive compound extraction

Polyphenolic compounds

For the extraction of bioactive compounds, 10 g of fruit-derived product (three replications) were put into a 50-mL test tube and 25 mL of extraction solution (methanol: bi-distilled water, 95:5 v/v, pH adjusted with 1.5 mL of 37% HCl) were then added to the weighed samples. After 60 min in the dark, the extracts were homogenized with an Ultra-Turrax (IKA-Werkemodel T25, Staufen,Germany) for about 1 min and then centrifuged for 15 min at 3,000 rpm in a Centrifuge (ALC

Centrifuge model PK 120, Cologno Monzese, Italy). This operation was carried out 3 times. All the supernatants were recovered and transferred to small glass tubes and kept frozen at -20°C for further analysis [1].

Organic acids, sugars, and monoterpenes

For the extraction of organic acids, sugars, and monoterpenes three replications were considered. Five grams of fruit jam/jelly were put into a test tube and 25 mL of 95% ethanol solution were then added. After 30 min in the dark, the extracts were homogenized with an Ultra-Turrax (T25, IKA WERKE) for about 1 min and then centrifuged for 10 min at 4,000 rpm in an ALC Centrifuge PK 120 (ALC International, Cologno Monzese, Italy). This operation was carried out 2 times. All the supernatants were recovered and transferred to small glass tubes and kept frozen at -20°C for further analysis [2].

Vitamic C

A total of 10 g of fruit-derived product (three replications) was put into a 50-mL test tube and 10 mL of extraction solution (0.1 M citric acid, 2 mM EDTA disodium salt and 4 mM sodium fluoride in methanol – water, 5:95 v/v) were then added. The extracts were homogenized with an Ultra-Turrax (IKA-Werke T25) for about 1 min and then centrifuged for 10 min at 4,000 rpm at room temperature in an ALC Centrifuge PK 120. The supernatants were recovered and transferred to a 15-mL test tube through filter cloth and then acidified with 4 N HCl to decrease pH solution to a value of 2.2–2.4 pH units. Acidified samples were centrifuged for 5 min at 12,000 rpm at 4°C with an ALC Multispeed refrigerated centrifuge PK 121R (ALC International, Cologno Monzese, Italy) [3].

Spectrophotometric analysis

The total polyphenol content (TPC) was determined by the Folin-Ciocalteu method [4,5], according to which the Folin-Ciocalteu reagent (2.5 mL) and 15% Na_2CO_3 (10 mL) were added to the extract (0.5 g), which was previously diluted with deionised water (30 mL). After 120 min, the absorbance was measured at 765 nm. The content of total phenolics was expressed as mg of gallic acid equivalents (GAE) per 100 g of product (Pr). The standard calibration curve was plotted using gallic acid at concentrations of 0.02–0.1 mg/mL. To evaluate the effect of sugar and citric acid added to the jams/jellies, a blank containing 65% sugar and 0.02% citric acid was used, although no correction was necessary at the dilution level used.

The total anthocyanin content (TAC) was evaluated by the pH differential method of Lee *et al.* [6],Giusti and Wrolstad [7], and expressed as milligrams of cyanidin 3-O-glucoside (C3G) per 100 grams of product (mg_{C3G}/100 g_{Pr}). A molar absorptivity of 26900 was used for cyanidin 3-glucoside (molecular weight 449.2). Phenolic extracts of jams/jellies in 0.025 M potassium chloride buffer (pH 1.0) and 0.4 M sodium acetate buffer (pH 4.5) were measured at 510 and 700 nm after 20 min of incubation at 23°C. Anthocyanins demonstrate maximum absorbance at 515 nm at pH 1.0 and also at 700 nm at pH 4.5. The colored oxonium form of anthocyanin predominates at pH 1.0, and the colorless hemiketal form at pH 4.5: the pH-differential method is based on the reaction producing oxonium forms.

For the evaluation of antioxidant activity the ferric reducing antioxidant power (FRAP) assay [8] was used and results were expressed as millimoles of ferrous iron (Fe^{2+}) equivalents per kilogram of Pr. The method is based on the reduction of the ferric (Fe^{3+}) 2,4,6-tris(2-pyridyl)-s-triazine (TPTZ) complex to its ferrous form (Fe^{2+}). The FRAP reagent was prepared daily by mixing a TPTZ solution and a $FeCl_3 \cdot 6H_2O$ solution with acetate buffer (0.3 M), and then warmed at 37°C before using; 30 μL of sample (15 μL of extracted sample and 15 μL of extraction buffer, dilution 1:2) was added to 90 μL of bi-distilled water and 900 μL of FRAP reagent in a 2-mL microtube and then incubated at 37°C for 30 min in a Shaking Water Bath (G.F.L. Shaking Water Bath mod.1083, Burgwedel,

Germany). Absorbance was read at 595 nm. Standard curve was obtained using $FeSO_4 \cdot 7H_2O$ (concentration range: 100–1,000 mmol/L).

An UV/Vis spectrophotometer (1600-PC, VWR International, Milan, Italy) single beam spectrophotometer was used to analyse TPC, anthocyanins, and antioxidant activity.

Chromatographic analysis

Sample preparation protocols for HPLC analysis

Samples were filtered with circular pre-injection filters (0.45 µm, polytetrafluoroethylene membrane) prior to HPLC-DAD analysis. In the case of vitamin C analysis, a C_{18} cartridge for solid phase extraction (Sep-Pak® C-18, Waters, Milford, MA, USA) was used to separate the polyphenolic fraction from vitamin C (ascorbic acid – AA plus dehydroascorbic acid – DHAA). Then, o-phenylenediamine (OPDA) solution (18.8 $mmol \cdot L^{-1}$) was added to each sample for DHAA derivatization into the fluorophore 3-(1,2-dihydroxyethyl)furo(3,4-b)quinoxaline-1-one. After 37 min in the dark, these samples were analyzed using HPLC-DAD [3].

Apparatus and chromatographic conditions

Separation and identification of compounds were performed by HPLC analysis, using an Agilent 1200 High-Performance Liquid Chromatograph coupled to an Agilent UV-Vis diode array detector (Agilent Technologies, Santa Clara, CA, USA).

Chromatographic separation were performed on a Kinetex – C18 column (4.6 × 150 mm, 5 µm, Phenomenex, Torrance, CA, USA), and a SphereClone – NH2 column (4.6 x 250 mm, 5 µm, Phenomenex, Torrance, CA, USA). Different chromatographic conditions were used to analyse the samples according to the methods described by [9], with some modifications.

The chromatographic conditions were set in order to obtain a phytochemical fingerprint containing compositional information with a good resolution and a reasonable analysis time. Different linear gradients in different slopes were used for optimizing the molecule separation because some

compounds were similar in structure with each other in the same chemical class. Formic and phosphoric acid was added for enhancing the resolution and eliminating peak tailing because most of the compounds were also weakly acidic. Detection was performed with an UV – Vis Diode Array Detector by scanning from 190 to 400 nm: selected wavelengths were suitable to achieve more specific peaks as well as a smooth baseline after a full-scan on the chromatogram.

Identification and quantification of bioactive compounds in the extracts

Identification of compounds was carried out by comparing retention times and spectroscopic data with those of authentic standards in the same chromatographic conditions. Total bioactive compound content (TBCC) was determined as the sum of selected markers having a positive role in human health as reported in the "multi-marker approach" by Mok and Chau [10]. Five polyphenolic classes were considered: benzoic acids (ellagic acid, gallic acid), catechins ((+)catechin, (-)epicatechin), cinnamic acids (caffeic acid, chlorogenic acid, coumaric acid, ferulic acid), flavonols (hyperoside, isoquercitrin, quercetin, quercitrin, rutin), and tannins (castalagin, vescalagin). Vitamin C (as the sum of ascorbic and dehydroascorbic acids), monoterpenes (limonene, phellandrene, sabinene, γ-terpinene, and terpinolene), organic acids (citric acid, malic acid, oxalic acid, quinic acid, succinic acid, and tartaric acid), and sugars (fructose, glucose, and sucrose) were also considered to obtain an analytical fingerprint. The total sugar content was calculated as the sum of fructose, glucose, and sucrose identified and quantified in the final products. Values include the initial total fruit sugar content and the sucrose added during the jam preparation. All the results were expressed as mg/100 g of Pr (product), except sugars (expressed as g/100 g of Pr).

External standard calibration method was used for quantitative determinations by plotting the peak area (y) of the compound versus the sample concentration (x): for each analytical standard three manual injections (20 μL) at each concentration were performed. The limit of detection (LOD) and the limit of quantification (LOQ) of the used chromatographic methods was defined as the lowest amount of analyte that gives a reproducible peak with a signal to-noise ratio (S/N) of 3 and 10

respectively. All the samples were analyzed in triplicate, and the results were reported as means ± standard deviation (SD) in order to assess the repeatability of the used methods. Accuracy was checked by spiking samples with a solution containing each bioactive compound in a concentration up to a maximum of 1.0 mg·mL^{-1}.

Statistical analysis

In order to establish the statistical differences between means the data were treated by one-factor analysis of variance (ANOVA), and the averages were compared with Tukey's HSD post-hoc comparison test at significance level $P < 0.05$ (N = 3). Correlation between antioxidant activity and TPC/TAC was evaluated with Pearson's coefficient (R) at $P < 0.05$ (N = 3).

Multivariate analysis (MVA) was carried out on all of the samples. The data matrix was defined as 24 objects (three repetitions for eight samples) and 12 variables (content of 9 chemical classes, TPC, TAC, antioxidant activity). Data were mean centred before MVA. Z-score scaling was subsequently carried out in order to check possible differences in the results. For discrimination of the investigated samples, principal component analysis (PCA) was performed on the column-centred data. All calculations were performed with statistical software package IBM SPSS Statistics 22.0 (IBM, Armonk, NY, USA).

Suppl. Figure 1. HPLC fingerprint of detected compounds in orange jams

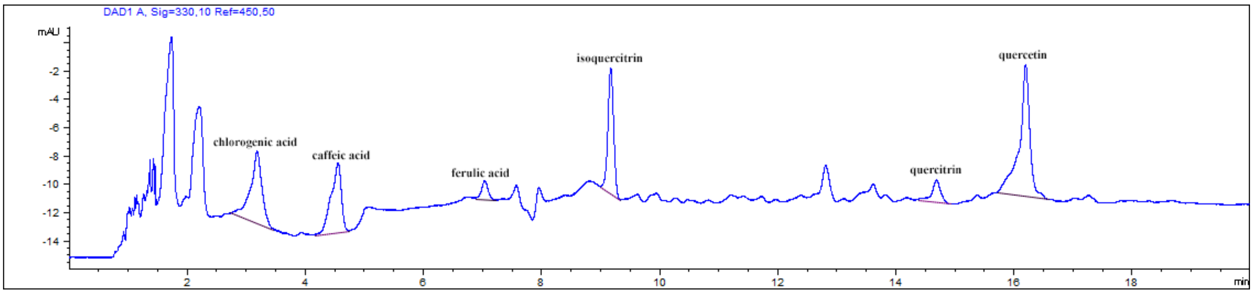

*cinnamic acids and flavonols

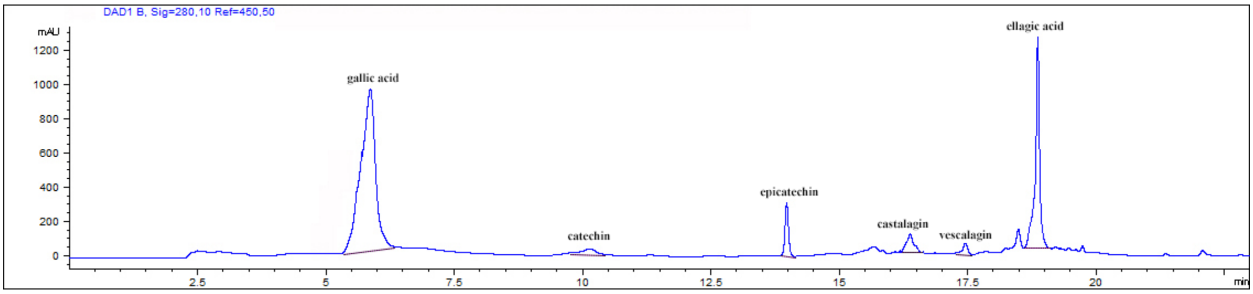

*benzoic acids, catechins, and tannins

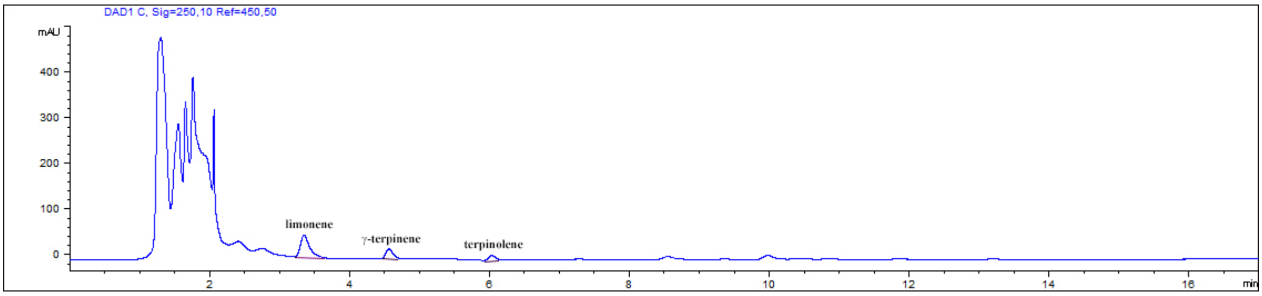

*monoterpenes

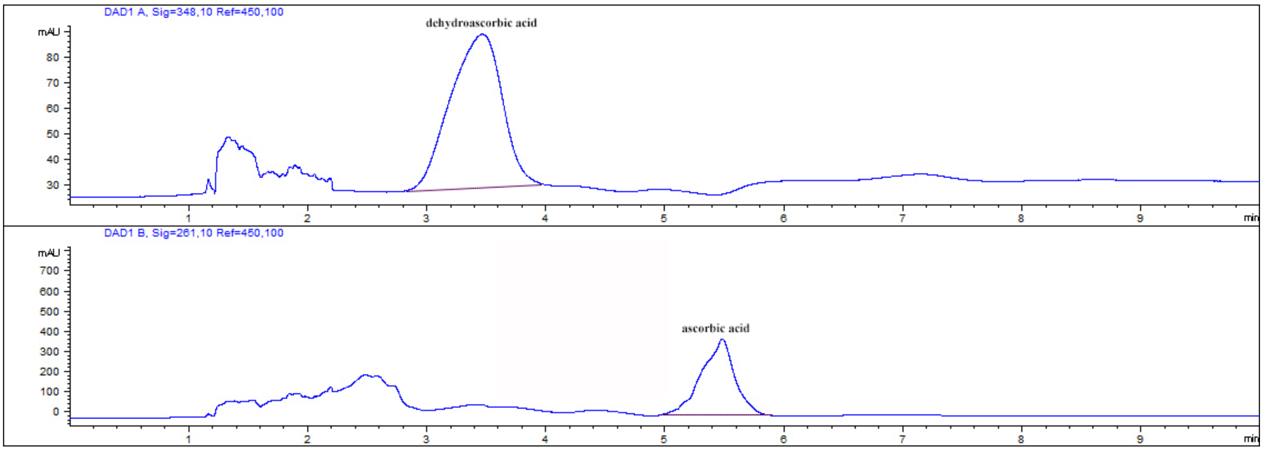

*vitamin C

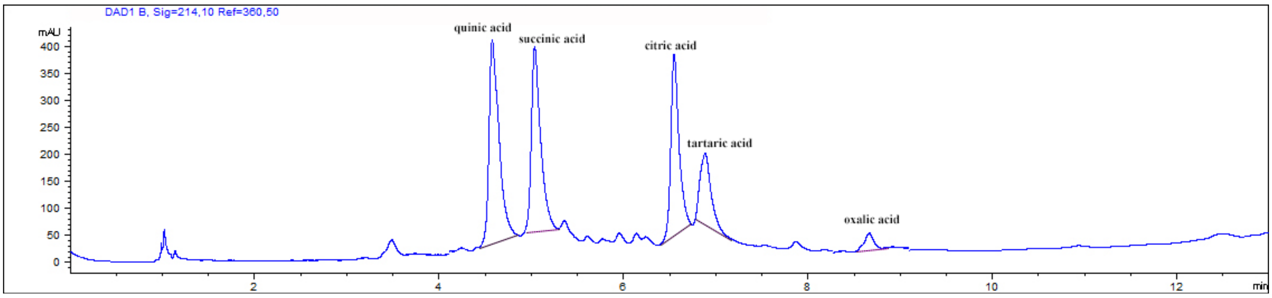

*organic acids

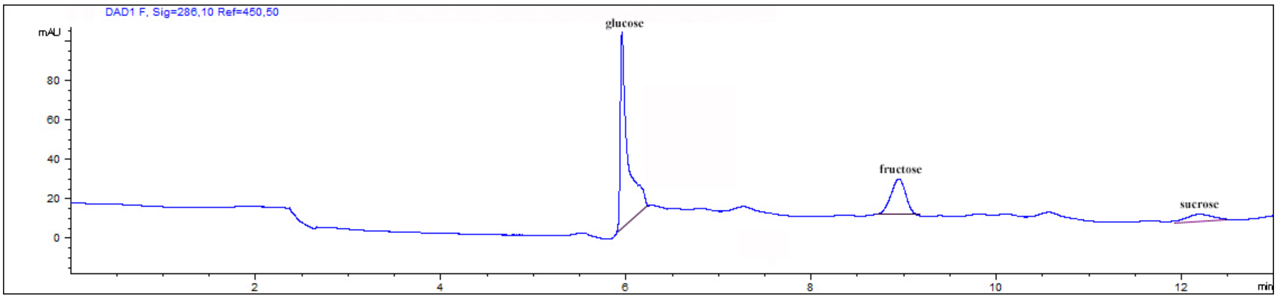

*sugars

REFERENCES

1. Donno, D.; Mellano, M.G.; De Biaggi, M.; Riondato, I.; Rakotoniaina, E.N.; Beccaro, G.L. New findings in *Prunus padus* L. fruits as a source of natural compounds: Characterization of metabolite profiles and preliminary evaluation of antioxidant activity. *Molecules (Basel, Switzerland)* **2018**, *23*.

2. Donno, D.; Beccaro, G.L.; Mellano, M.G.; Cerutti, A.K.; Bounous, G. Goji berry fruit (*Lycium* spp.): Antioxidant compound fingerprint and bioactivity evaluation. *J. Func. Foods* **2015**, *18, Part B*, 1070-1085.

3. Gonzalez-Molina, E.; Moreno, D.A.; Garcia-Viguera, C. Genotype and harvest time influence the phytochemical quality of fino lemon juice (*Citrus limon* (L.) Burm. F.) for industrial use. *J. Agric. Food Chem.* **2008**, *56*, 1669-1675.

4. Slinkard, K.; Singleton, V.L. Total phenol analysis: Automation and comparison with manual methods. *Am. J. Enol. Vitic.* **1977**, *28*, 49-55.

5. Sánchez-Rangel, J.C.; Benavides, J.; Heredia, J.B.; Cisneros-Zevallos, L.; Jacobo-Velázquez, D.A. The Folin–Ciocalteu assay revisited: Improvement of its specificity for total phenolic content determination. *Anal. Methods* **2013**, *5*, 5990-5999.

6. Lee, J.; Durst, R.W.; Wrolstad, R.E. Determination of total monomeric anthocyanin pigment content of fruit juices, beverages, natural colorants, and wines by the pH differential method: Collaborative study. *J. AOAC Int.* **2005**, *88*, 1269-1278.

7. Giusti, M.M.; Wrolstad, R.E. Characterization and measurement of anthocyanins by UV-visible spectroscopy. In *Current protocols in food analytical chemistry*, John Wiley & Sons, Inc., 2001.

8. Benzie, I.F.; Strain, J.J. Ferric reducing/antioxidant power assay: Direct measure of total antioxidant activity of biological fluids and modified version for simultaneous measurement of total antioxidant power and ascorbic acid concentration. *Meth. Enzymol.* **1999**, *299*, 15-27.

9. Donno, D.; Mellano, M.G.; Prgomet, Z.; Beccaro, G.L. Advances in *Ribes* x *nidigrolaria* Rud. Bauer and A. Bauer fruits as potential source of natural molecules: A preliminary study on physico-chemical traits of an underutilized berry. *Sci. Hortic.* **2018**, *237*, 20-27.

10. Mok, D.K.W.; Chau, F.T. Chemical information of chinese medicines: A challenge to chemist. *Chemometrics Intell. Lab. Syst.* **2006**, *82*, 210-217.
